# Trisubstituted-Imidazoles Induce Apoptosis in Human Breast Cancer Cells by Targeting the Oncogenic PI3K/Akt/mTOR Signaling Pathway

**DOI:** 10.1371/journal.pone.0153155

**Published:** 2016-04-20

**Authors:** Chakrabhavi Dhananjaya Mohan, V. Srinivasa, Shobith Rangappa, Lewis Mervin, Surender Mohan, Shardul Paricharak, Sefer Baday, Feng Li, Muthu K. Shanmugam, Arunachalam Chinnathambi, M. E. Zayed, Sulaiman Ali Alharbi, Andreas Bender, Gautam Sethi, Basappa, Kanchugarakoppal S. Rangappa

**Affiliations:** 1 Department of Studies in Chemistry, Manasagangotri, University of Mysore, Mysore 570006, India; 2 Laboratory of Chemical Biology, Department of Chemistry, Bangalore University, Central College Campus, Palace Road, Bangalore 560001, India; 3 Frontier Research Center for Post-Genome Science and Technology, Hokkaido University, Sapporo 060–0808, Japan; 4 Centre for Molecular Informatics, Department of Chemistry, University of Cambridge, Lensfield Road, Cambridge, United Kingdom; 5 Laboratory of Molecular Biology and Genetic Engineering, School of Biotechnology, Jawaharlal Nehru University, New Delhi 110067, India; 6 Division of Medicinal Chemistry, Leiden Academic Centre for Drug Research, Leiden University, P.O. Box 9502, 2300 RA Leiden, The Netherlands; 7 Applied Informatics Department, Informatics Institute, Istanbul Technical University, 34469, Istanbul, Turkey; 8 Department of Pharmacology, Yong Loo Lin School of Medicine, National University of Singapore, Singapore, Singapore; 9 Department of Botany and Microbiology, College of Science, King Saud University, Riyadh -11451, Kingdom of Saudi Arabia; 10 School of Biomedical Sciences, CHIRI Biosciences Research Precinct, Curtin University, Western Australia 6009, Australia; Universidade Federal do Rio de Janeiro, BRAZIL

## Abstract

Overactivation of PI3K/Akt/mTOR is linked with carcinogenesis and serves a potential molecular therapeutic target in treatment of various cancers. Herein, we report the synthesis of trisubstituted-imidazoles and identified 2-chloro-3-(4, 5-diphenyl-1H-imidazol-2-yl) pyridine (CIP) as lead cytotoxic agent. Naïve Base classifier model of *in silico* target prediction revealed that CIP targets RAC-beta serine/threonine-protein kinase which comprises the Akt. Furthermore, CIP downregulated the phosphorylation of Akt, PDK and mTOR proteins and decreased expression of cyclin D1, Bcl-2, survivin, VEGF, procaspase-3 and increased cleavage of PARP. In addition, CIP significantly downregulated the CXCL12 induced motility of breast cancer cells and molecular docking calculations revealed that all compounds bind to Akt2 kinase with high docking scores compared to the library of previously reported Akt2 inhibitors. In summary, we report the synthesis and biological evaluation of imidazoles that induce apoptosis in breast cancer cells by negatively regulating PI3K/Akt/mTOR signaling pathway.

## Introduction

PI3K/Akt/mTOR pathway is a major signaling cascade which operates downstream to the receptor tyrosine kinases such as epidermal growth factor receptor (EGFR), platelet-derived growth factor receptor (PDGFR) and insulin-like growth factor-1 receptor (IGF-1R) [1–3]. The relay of signals from the aforementioned growth factor receptors leads to the activation of Phosphoinositide 3-kinase (PI3K) which catalyses the generation of phosphatidylinositol-3,4,5-triphosphate (PIP3) from phosphatidylinositol-4,5-biphosphate [4]. In turn, PIP3 interacts with pleckstrin homology (PH) domains of phosphoinositide-dependent kinase (PDK) and Akt (Also called as Protein Kinase B) and activates them [5]. In addition, PDK contributes to activation of Akt. Among these two master kinases, Akt acts on wide array of substrates which are involved in regulation of apoptosis, cell cycle, transcription and translation [6, 7]. Therefore, the PI3K/Akt/mTOR pathway plays a central role in regulation of the cell proliferation, survival, migration, angiogenesis and metabolism and extensively contributes to oncogenesis [8–11]. Overactivation of PI3K/Akt/mTOR pathway has been reported in many types of cancers offering a unique therapeutic target to design novel heterocycles against malignancies [12–15].

Imidazole-based compounds have been extensively studied and have been reported to possess good anticancer activity in various types of cancer cells [16, 17]. Previously, we reported the synthesis and antiproliferative activity of imidazole derivatives in various tumor models [18–20]. Studies have suggested that imidazole conjugates possess good PI3K inhibitory activity [21]. SB203580 is a pyridinyl imidazole reported to effectively inhibit PI3K indicate that imidazole-based compounds are pharmacologically important scaffolds to target different type of cancers [21]. Therefore, in continuation our effort to synthesize and explore the various pharmacological properties of heterocycles [22–29], in the present study, we synthesized a series of trisubstituted imidazole derivatives and evaluated their inhibitory efficacy against PI3K/Akt/mTOR signaling pathway in breast cancer cell lines. An *in silico* target prediction revealed that new compounds target RAC-beta serine/threonine-protein kinase which comprise the Akt and abrogates PI3K/Akt/mTOR pathway. The lead compound, 2-chloro-3-(4, 5-diphenyl-1H-imidazol-2-yl) pyridine (CIP) suppressed the proliferation of breast cancer cells, decreased the phosphorylation of PDK, Akt, mTOR, downregulated the cellular invasion and activated caspases and cleaved PARP to induce apoptosis.

## Materials and Methods

All solvents used were of analytical grade and reagents used were purchased from Sigma-Aldrich. ^1^H and ^13^C NMR spectra were recorded on a Agilent (400 MHz) spectrometer in CDCl_3_ or DMSO-d_6_ as solvent, using TMS as an internal standard and chemical shifts are expressed as ppm. Mass and purity were recorded on a LC-MSD-Trap-XCT. High resolution mass spectra were determined on a Bruker Daltonics instrument. The progress of the reaction was monitored by TLC pre-coated silica gel G plates. The breast cancer MCF-7 and MDA-MB-231 cell lines were obtained from ATCC (Manassas, VA, USA).

### General procedure for the synthesis of the trisubstituted imidazoles

**Fig 1:**
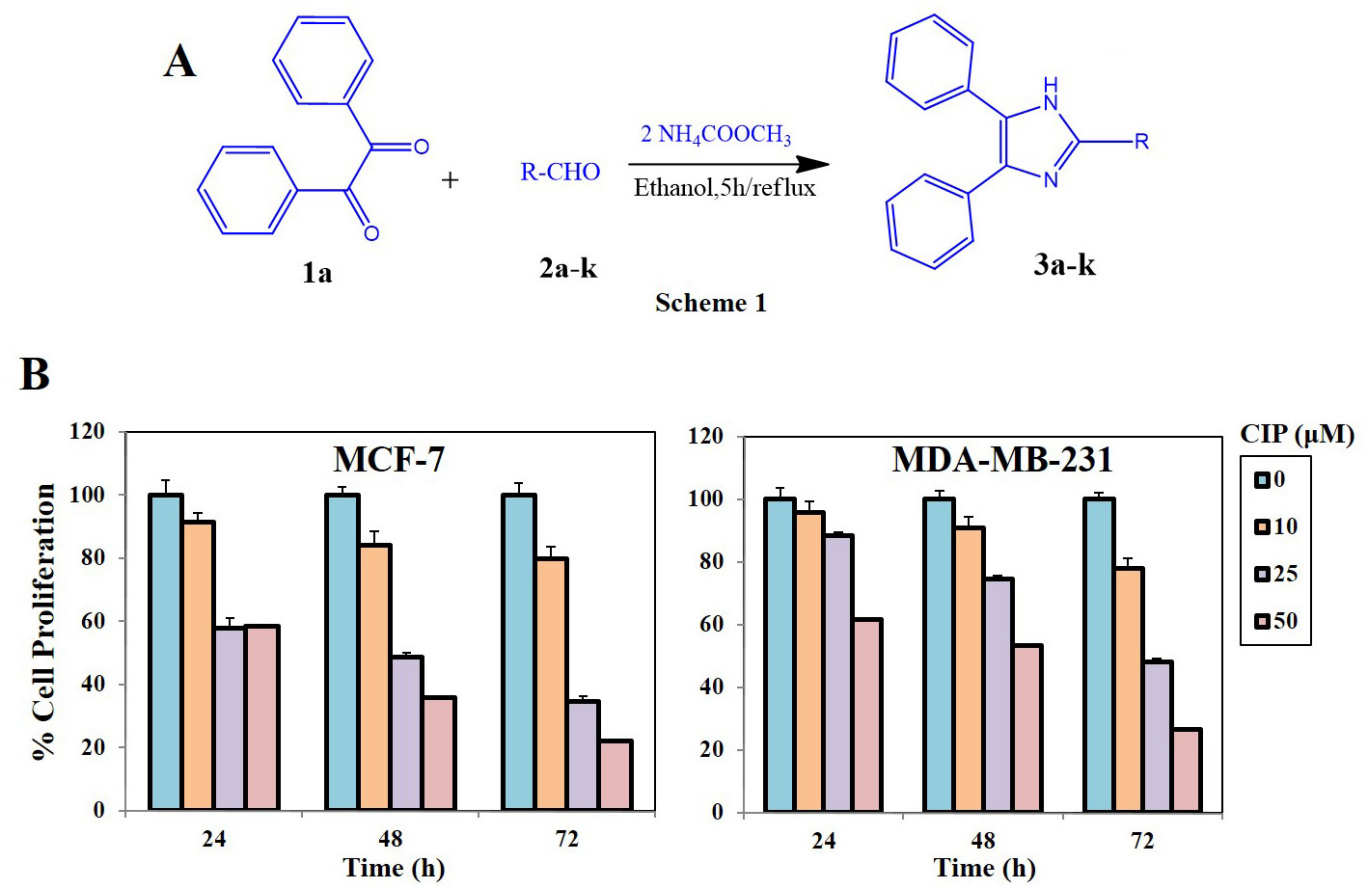
A, Schematic representation for the synthesis of imidazole based small molecules. B, Breast cancer cells (2.5 X 10^4^/mL, MCF-7 & MDA-MB-231) were plated in triplicate, treated with indicated concentrations (0, 10, 25 and 50 μM) of CIP, and then subjected to MTT assay after 24, 48 and 72 h to analyse proliferation of cells. CIP suppresses the viability of various breast cancer cell lines in a dose- and time-dependent manner.

The reaction mixture of benzil (1.0 mmol), aldehydes (1.0 mmol), NH_4_OAc (4.0 mmol) and a metal halide catalyst (0.10 mmol), followed by EtOH (4 ml) was stirred and gently refluxed for 5 h (Fig 1A). The completion of reaction was monitored by TLC and 4 ml of water was added at the end of reaction. The solid was filtered, washed with a mixture of cold EtOH/H_2_O (1:1, v:v). The crude product was recrystallized from acetone/water 9:1.

#### 2-(3, 4-Dimethoxyphenyl)-4, 5-diphenyl-1H-imidazole (1)

^1^H NMR (CDCl_3_, 400 MHz) δ: 12.454 (s, NH), 6.878–7.631 (m, 13H), 3.961 (s, 6H). ^13^C NMR: 149.7, 148.9, 147.9, 136.7, 134.9, 131.4, 131.3, 129.9, 129.3, 129.1, 128.2, 126.6, 126.4, 123.7, 122.1, 113.2, 111.7, 55.7, 55.6. (MM—ES + APCI) m/z: 357.12 [M+H] ^+^. MP 218–219°C

#### 2-Bromo-5-(4, 5-diphenyl-1H-imidazol-2-yl) pyridine (2)

^1^H NMR (CDCl_3_, 400 MHz) δ: 13.205(s, NH), 8.777–8.761 (d, J = 4 Hz, 1H), 8.141–8.111 (dd, J_1_ = 4 Hz, J_2_ = 4 Hz, 1H), 7.483–7.318 (m, 5H), 7.314–7.241 (m, 6H). ^13^C NMR: 148.9, 145.3, 141.9, 134.9, 130.4, 130. 5, 129.9, 129. 5, 129.1, 128.2, 126. 9, 126. 3, 123.7, 122.2. (MM—ES + APCI) m/z: 377.04 [M+H] ^+^, HRMS (ESI–TOF) Calculated for C_20_H_14_N_3_Br [M + Na], 398.0263: found 398.0266. MP 248–249°C

#### 2-(4-(Methylsulfonyl) phenyl)-4, 5-diphenyl-1H-imidazole (3)

^1^H NMR (CDCl_3_, 400 MHz) δ: 13.092 (s, NH), 8.391–8.371 (d, J = 8 Hz, 2H), 8.095–8.075 (d, J = 8 Hz, 2H), 7.596–7.377 (m, 10H), 3.391 (s, 3H). ^13^C NMR: 147.6, 146.9, 131.8, 131.7, 129.8, 129.7, 129.5, 128.9, 126.9, 126.8 123.7, 122.1, 113.2, 111.7, 55.4. (MM-ES+APCI) m/z: 375.11 [M+H] ^+^, HRMS (ESI–TOF) Calculated for C_22_H_18_N_2_O_2_S [M+Na], 397.0981: found 397.0983. MP 256–257°C

#### 2-(4-Butoxyphenyl)-4, 5-diphenyl-1H-imidazole (4)

^1^H NMR (CDCl_3_, 400 MHz) δ: 13.20 (s, NH), 8.095–8.075 (d, J = 8 Hz, 2H), 7.923–7.765 (m, 2H) 7.483–7.324 (m, 10H), 3.941–3.867 (m, 2H), 2.171–1.322 (m, 6H). ^13^C NMR: 146.9, 137.6, 133.7, 129.5, 129.5, 129.4, 128.9, 126.5, 126.0, 124.7 122.7, 114.7, 65.6, 31.8, 24.1, 17.18. (MM—ES + APCI) m/z: 369.17 [M+H] ^+^. MP 229–230°C

#### 3-(4, 5-Diphenyl-1H-imidazol-2-yl)-1H-indole (5)

^1^H NMR (CDCl_3_, 400 MHz) δ: 12.4 (s, 1H), 11.404 (s, 1H), 8.461–8.441 (d, J = 8 Hz, 1H), 8.016–8.006 (d, J = 4 Hz, 1H), 7.587–7.128 (m, 13H). ^13^C NMR: 144.1, 136.7, 128.9, 128.5, 127.6, 125.1, 124.5, 122.4, 121.9, 120.4, 112.1, 106.9. (MM-ES+APCI) m/z: 336.47 [M+H]^+^. MP 290–291°C

#### 3-(4, 5-Diphenyl-1H-imidazol-2-yl)-2-methyl-1H-indole (6)

^1^H NMR (CDCl_3_, 400 MHz) δ: 11.553 (s, 1H, NH), 10.07 (s, 1H, NH), 8.147–7.484 (m, 4H), 7.419–7.102 (m, 10H), 2.214 (s, 3H). ^13^C NMR: 143.9, 136.7, 136.4, 134.98, 132.6, 128.8, 128.5, 127.6, 126.8, 126.4, 125.69, 125.31, 124.7, 122.6, 121.8, 120.7, 110.6, 102.8, 14.71. (MM-ES+APCI) m/z: 350.16 [M+H] ^+^. MP 298–299°C.

#### 4, 5-Diphenyl-2-(thiophen-2-yl)-1H-imidazole (7)

^1^H NMR (CDCl_3_, 400 MHz) δ: 12.79 (s, 1H, NH), 7.15–7.13 (dd, J_1_ = 4 Hz, J_2_ = 4 Hz, 1H), 7.69–7.68 (d, J = 4 Hz, 1H), 7.51–7.25 (m, 11H). ^13^C NMR: 141.5, 136.6, 134.7, 133.9, 130.8, 128.6, 128.1, 127.8, 127.0, 126.5, 124.1. (MM-ES+APCI) m/z: 303.37 [M+H] ^+^. MP 255–256°C

#### 4, 5-Diphenyl-2-(thiophen-3-yl)-1H-imidazole (8)

^1^H NMR (CDCl_3_, 400 MHz) δ: 12.63 (s, 1H, NH), 8.04–8.02 (dd, J_1_ = 4 Hz, J_2_ = 4 Hz, 1H), 7.71–7.68 (dd, J_1_ = 4Hz, J_2_ = 4 Hz, 1H), 7.65–7.63 (dd, J_1_ = 4 Hz, J_2_ = 4 Hz, 1H), 7.31–7.53 (m, 10H), ^13^C NMR: 121.8, 124.2, 126.5, 127.0, 127.7, 128.1, 128.4, 130.8, 132.5, 134.7, 136.6, 142.7 (MM—ES + APCI) m/z: 303.37 [M+H] ^+^. MP 257–259°C

#### 2-Chloro-3-(4, 5-diphenyl-1H-imidazol-2-yl) pyridine (9, CIP)

^1^H NMR (CDCl_3_, 400 MHz) δ: 12.591 (1H, NH), 8.841–8.811 (dd, J_1_ = 4 Hz, J_2_ = 4 Hz, 1H), 8.390–8.370 (dd, J_1_ = 4 Hz, J_2_ = 4 Hz, 1H), 7.567–7.560 (m, 4H), 7.406–7.307 (m, 7H). ^13^C NMR: 148.7, 140.1, 139.9, 133.9, 130.4, 131. 0, 129.9, 122.2, 129. 5, 129.1, 128.2, 126. 9, 126. 3, 123.7, (MM-ES+APCI) m/z: 332.09 [M+H]^+^, HRMS (ESI–TOF) Calculated for C_20_H_14_ClN_3_ [M + Na], 354.0768: found 354.0766. MP 228–229°C

#### 2-(4-Chloro-1, 3-dimethyl-2, 5-dihydro-1H-pyrazol-5-yl)-4, 5-diphenyl-1H-imidazole (10)

^1^H NMR (CDCl_3_, 400 MHz) δ: 12.591(1H, NH), 7.571–7551 (m, 4H), 7.352–7.283 (m, 6H), 4.348 (s, 1H), 3.829 (s, 3H), 2.609 (s, 3H), 2.254 (s, 1H). ^13^C NMR: 149.7, 140.3, 139.9, 134.9, 130.4, 131. 0, 129.9, 129. 5, 129.1, 128.2, 126. 9, 76. 3, 43.7, 12.2, (MM-ES+APCI) m/z: 351.17 [M+H]^+^, HRMS (ESI–TOF) Calculated for C_20_H_19_ClN_4_ [M+Na], 373.1190: found 373.1193. MP 218–219°C.

#### 2-(Naphthalen-2-yl)-4, 5-diphenyl-1H-imidazole (11)

^1^H NMR (CDCl_3_, 400 MHz) δ: 12.71 (s, NH), 8.69 (s, 1H), 8.26–8.24 (d, J = 8 Hz, 2H), 7.967–7.501 (m, 7H), 7.498–7.284 (m, 7H). ^13^C NMR: 145.4, 137.9, 137.0, 135.2, 133.5, 130.9, 130.2, 129.4, 128.7, 128.4, 128.2, 128.0, 127.7, 127.5, 127.3, 127.0, 126.5, 126.4, 126.3, 125.9, 125.0 (MM—ES + APCI) m/z: 347.20 [M+H]^+^. MP 273–275°C.

### Pharmacology

#### MTT Assay

The antiproliferative effect of newly synthesized compounds against breast cancer cells was determined by the MTT dye uptake method as described previously [30]. The breast cancer MCF-7 and MDA-MB-231 cell lines were obtained from ATCC (Manassas, VA, USA). Briefly, breast cancer cells (2.5 X 10^4^/ml) were incubated in triplicate in a 96-well plate, in the presence of varying compound concentrations at a volume of 0.2 ml, for different time intervals at 37°C. Thereafter, a 20 μl MTT solution (5 mg/ml in PBS) was added to each well. After a 2 h incubation at 37°C, a 0.1 ml lysis buffer (20% SDS, 50% dimethylformamide) was added; incubation was performed for 1 h at 37°C, and the optical density (OD) at 570 nm was measured by Tecan plate reader. 0.01% DMSO was used as the negative control and 0.01% MTT was used as a control agent.

#### Flow cytometric analysis

Flow cytometric analysis was performed to determine whether CIP can induce apoptosis of tumor cells as described earlier [31]. Briefly, MDA-MB-231 breast cancer cells (5 x 10^5^) were plated in petri dish and 24 h later the cells were exposed to compound CIP (50 μM) for 0, 24, 36 and 48 h. Thereafter cells were washed, fixed with 70% ethanol, and incubated for 30 min at 37°C with 0.1% RNase A in PBS. Cells were washed again, resuspended, and stained with PBS containing 25 μg/ml propidium iodide (PI) for 15 min at room temperature. The cell cycle distribution across the various phases was analyzed with a CyAn ADP flow cytometer (Dako Cytomation).

#### Caspase 3/7 activity assay

MDA-MB-231 cells (5 x 10^5^) were plated in 6-well plates and allowed to adhere for 24 h. The cells were then exposed to CIP (50 μM) for 0, 24, 36 and 48 h. Thereafter, the Caspase-3/7 activities were determined by a Caspase-Glo^®^ 3/7 assay kit (Promega, Madison, USA) according to the manufacturer’s instructions.

#### Target prediction-based enrichment of Imidazole series

In order to understand the mode-of-action of trisubstituted imidazoles towards the inhibition of human breast cancer cells, we applied an *in silico* target prediction tool, which was developed using the Bernoulli Naïve Bayes (BNB) algorithm, as implemented in Scikit-learn [32]. This method has been validated previously by Mervin *et al* [33]. Active compounds were extracted from ChEMBL [34], using pChEMBL values larger than 5 (10 μM) and a confidence score cut-off of 5 to define activity. Inactive data points were extracted from PubChem [35] for compounds declared inactive from screens stored within BioAssay [36]. The BNB model was trained on these compounds using the 2048 bit Morgan fingerprints, with a radius of 2, generated by RDKit [37]. Stringent class-specific thresholds were applied to binarize predictions for the Imidazole series.

In order to improve the statistical significance of this analysis, predictions for the imidazole sets are compared to predictions generated by chance for 1,000 sets of randomly selected compounds from the PubChem repository. An *Average Ratio* enrichment metric is calculated from this analysis (Eq 1), which reflects the average of the series of predictions in the background (R_*i*_), divided by the frequency of predictions in the imidazole series (F_*imidazole*_). A lower score represents targets that are more enriched, and hence found to be significant for the imidazole series.

$$Average\;Ratio\;=\;\;\frac{1}{1,000}\;\sum_{i=1}^{1,000}\;\frac{Ri}{F\;imidazole}\;$$ (1)

#### Western blotting

Western blotting analysis was performed as described earlier [38]. Briefly, For detection of various proteins, CIP-treated whole-cell extracts were lysed in a lysis buffer (20 mM Tris (pH 7.4), 250 mM NaCl, 2 mM EDTA (pH 8.0), 0.1% TritonX-100, 0.01 mg/ml aprotinin, 0.005 mg/ml leupeptin, 0.4 mM PMSF,and 4 mM NaVO_4_). Lysates were then spun at 14,000 rpm for 10 mins to remove insoluble material, and then resolved on a 7.5% SDS gel. After electrophoresis, the proteins were electrotransferred to a nitrocellulose membrane, blocked with 5% non-fat milk, and probed with various antibodies (1:1000) overnight at 4°C. The blot was washed, exposed to HRP-conjugated secondary antibodies for 1 h, and finally examined by chemiluminescence (ECL; GE Healthcare, Little Chalfont, Buckinghamshire, UK).

#### Invasion Assay

A BD Biocoat^™^ Matrigel^™^ invasion chamber with 8-μm pores in the light-tight polyethylene terephthalate membrane and was coated with a reconstituted basement membrane gel (BD Biosciences). 2 X 10^5^ cells were suspended in serum-free DMEM and seeded into the Matrigel transwell chambers. The cells were incubated with CIP for 8 h. After incubation, the outer surfaces of the transwell chambers were wiped with cotton swabs, and the invading cells were fixed and stained with crystal violet solution. The invading cells were then counted in five randomly selected areas under microscopic observation.

#### Molecular docking analysis

The trisubstituted imidazoles were docked to the crystal structure of human RAC-Beta Serine/Threonine-Protein Kinase (Akt2) complexed with an inhibitor (PDB: 2JDR) [39] using MOE [40]. Protonation states of amino acids were assigned using protonate3D in MOE [41]. Ligands were ionized at physiological pH using MOE. The binding site was defined by the position of the co-crystalized ligand. Docking calculations were carried out using MOE’s induced fit protocol that treats amino acid side-chains near the binding site as flexible.

## Results and Discussion

### Chemical synthesis of trisubstituted imidazoles

We previously reported the microwave assisted synthesis and crystal structure studies of 2-butyl-4-chloro-imidazole-5-carbaldehyde [19, 20, 42]. In addition, solvent free and microwave-assisted synthesis of 4,5-disubstituted imidazoles have been reported using 1,2-diketones and urotropine in the presence of ammonium acetate [43]. In this report, we prepared 1,2,4-trisubstituted-1H-imidazoles by heating a mixture of benzil, an aldehyde and ammonium acetate using ethanol as solvent. The obtained imidazoles (**3a-k**) were characterized by melting point, LC-MS, ^1^H NMR, and ^13^C NMR analysis. The appearance of a ^1^H NMR peak at δ value of 12.5–12.7 for the HN-proton of imidazole confirms the ring formation. Detailed physical parameters of all the compounds synthesized is provided as supplemental information (S1 Table).

### Trisubstituted imidazoles elicit an antiproliferative effect against human breast cancer cells

Imidazole derivatives are known for their antiproliferative effect towards various cancer models. We initially evaluated the effect of novel trisubstituted imidazoles against MDA-MB-231 cells using MTT assay as described earlier [44, 45]. Among the newly synthesized structures, compound 1, 2 and CIP displayed antiproliferative effect with the IC_50_ values of 21.1, 17.8 and 24.1 μM respectively. Further investigation of the compound 1, 2 and CIP against mammary epithelial cells (MCF-10A) revealed that, CIP selectively induce cytotoxicity in breast cancer cells and did not exhibit significant cytotoxicity against normal cells. To confirm the antiproliferative potential of the trisubstituted imidazoles, we further investigated the effect of compounds on hepatocellular carcinoma (HepG2) and normal (LO2) cells and found CIP as a lead anticancer compound with minimal cytotoxicity towards non-cancerous cells. The cytotoxicity profile of all the compounds is provided as supplementary information (S1 Table). Thereafter, we investigated the effect of CIP on MCF-7 and MDA-MB-231 breast cancer cells at different doses (0, 10, 25 & 50 μM) and time-points (24, 48 & 72 h). Upon treatment with CIP, we observed a significant decline in the viability of both the cell lines in a dose and time dependent manner (Fig 1B).

Investigation of structure-activity relationship (SAR) of the tested molecules suggests that introducing small sized and electron withdrawing atom containing heterocyclic groups at the position 2 in the imidazole ring would favour the specificity for cancer cells. Molecules having these SAR features better match the polarity of the Akt2 binding surface, since polar region in the Akt2 binding pocket is small.

### CIP significantly accumulates MDA-MB-231 cells in sub-G1 phase and increases the activity of executioner caspases

**Fig 2:**
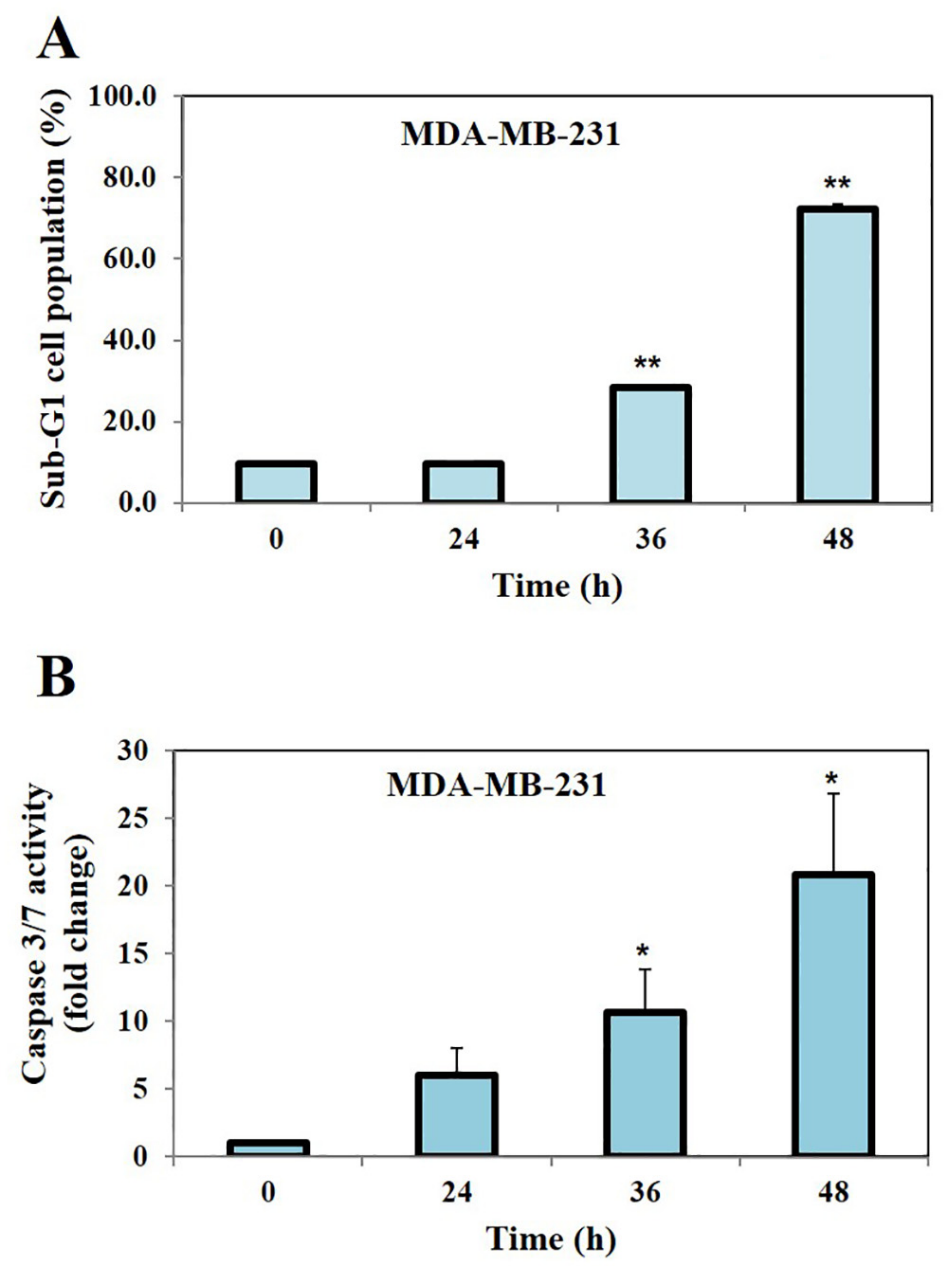
A, The analysis of cell cycle distribution following treatment with CIP was performed using flow cytometry. MDA-MB-231 cells were exposed to compound CIP (50 μM) for indicated time intervals (0, 24, 36 and 48 h), after which the cells were harvested and stained with PI. The cell distribution across the various phases of the cell cycle was analyzed with a flow cytometer. We observed that CIP induced significant apoptosis in a time dependent manner as evidenced by increased accumulation of cells in Sub-G1 phase of the cell cycle. B, MDA-MB-231 cells were exposed to compound CIP (50 μM) at indicated time intervals (0, 24, 36 and 48 h), after which they were harvested and caspase3/7 activity was measured using Caspase-Glo^®^ 3/7 assay kit. We found that treatment of MDA-MB-231 cells with CIP caused the significant increase in the caspases-3/7 activity. * for p<0.05, ** for p<0.005.

We next investigated the potential of CIP to induce apoptosis in breast cancer MDA-MB-231 cells. We first observed that CIP induced significant apoptosis in a time dependent manner as evidenced by increased accumulation of cells in Sub-G1 phase of the cell cycle (Fig 2A). We further noted that exposure of the CIP also caused a significant increase in the activity of effector caspases -3 and -7 (Fig 2B), thereby indicating the apoptotic potential of CIP against breast cancer cells.

### In silico mode-of-action analysis for CIP that inhibits the growth of human breast cancer cells

In order to understand the molecular mechanism of CIP responsible for the antiproliferative effect in human breast cancer cells, we carried out an *in silico* target prediction analysis. Enrichment for the targets from the *in silico* target prediction analysis are shown in supplementary table (S2 Table). The top 20 targets from the 1,080 models are shown. “RAC-Beta Serine/Threonine-Protein Kinase” (Ranked 2^nd^) and “RAC-Alpha Serine/Threonine-Protein Kinase” (Ranked 8^th^), comprise the Akt protein, relevant for this study due to its involvement in the PI3K/Akt/mTOR pathway, and are highly enriched in this analysis.

### CIP downregulates phosphorylation of PDK, Akt and mTOR in breast cancer cells

**Fig 3:**
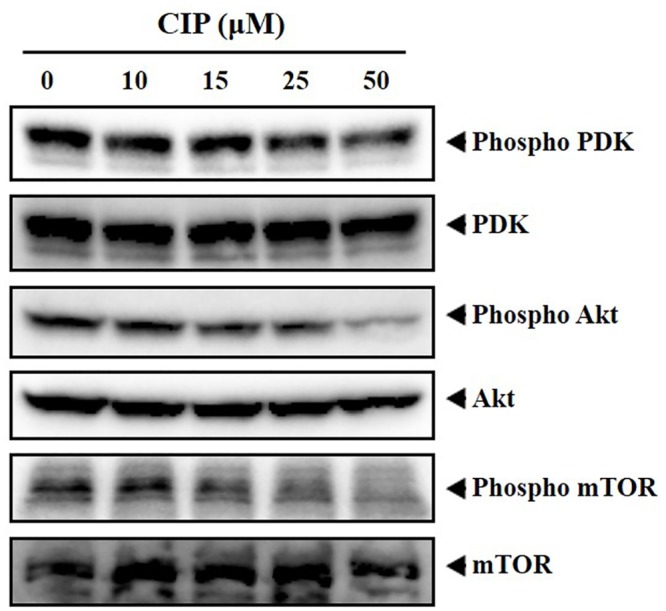
MDA-MB-231 cells were treated with CIP at indicated concentrations (0, 10, 15, 25 and 50 μM) for 8 h and levels of phospho-PDK, phospho-Akt, phospho-mTOR and PDK, Akt, mTOR was analysed using western blotting. We found that, CIP downregulated the phosphorylation of Akt, PDK and mTOR in a dose-dependent manner without any change in the expression levels of Akt, PDK and mTOR proteins.

Based on the *in silico* prediction, we next analysed whether CIP modulates phosphorylation of Akt, PDK and mTOR in MDA-MB-231 cells using western blotting as described previously [46, 47]. As observed by western blot analysis, we found that, CIP downregulated the phosphorylation of Akt, PDK and mTOR in a dose-dependent manner (Fig 3) and at the same time Akt, PDK and mTOR protein expression remained unaltered.

### CIP induces apoptosis of human breast cancer cells

**Fig 4:**
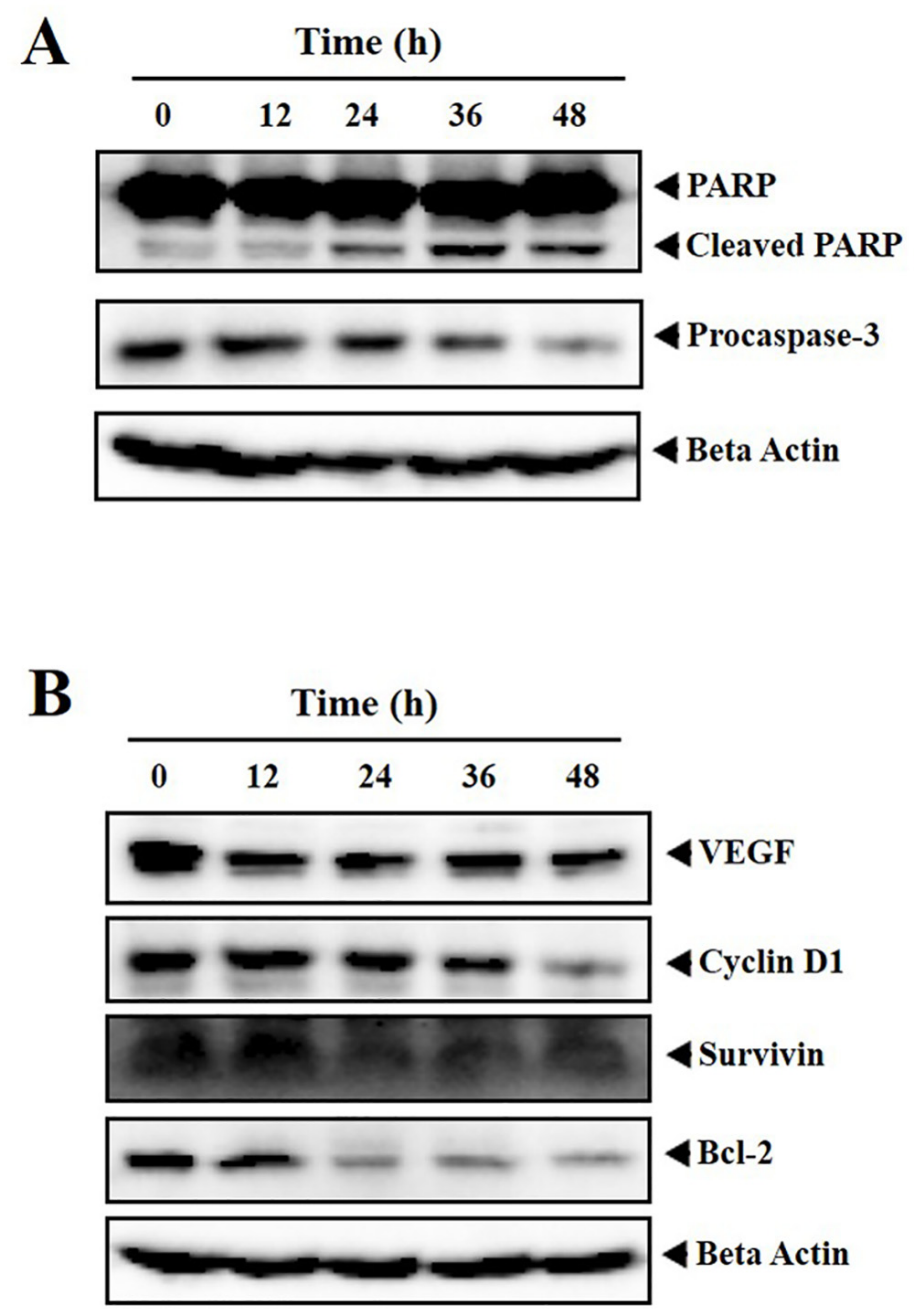
A and B, MDA-MB-231 cells were treated with CIP (25 μM) for indicated time intervals (0, 12, 24, 36 and 48 h) and expression of apoptotic markers (PARP, Procaspase-3), antiapoptotic proteins (Bcl-2, Survivin, VEGF), cell cycle regulator (Cyclin D1) was profiled using western blot analysis. We observed the significant decline in the expression of PARP, procaspase-3, cyclin D1, VEGF, survivin and Bcl-2 proteins in the time-dependent manner without alteration in the levels of beta actin.

Cell shrinkage, formation of apoptotic bodies, activation of caspases, and cleavage of chromosomal DNA mediated by caspase dependent nucleases are the major events in the cells undergoing apoptosis [48]. In the event of cell committed to apoptosis, cascade of reactions occurs ultimately leading to the activation of caspase-3 which further cleaves full length PARP into fragments [49, 50]. Therefore, we first investigated the effect of CIP on procaspase-3 and found the activation of procaspase-3 in a time time-dependent manner (Fig 4A). Similarly, we observed the subsequent decrease in full length PARP with increase in cleaved fragment. We further determined the effect of CIP on the expression of various proliferative and survival proteins such as cyclin D1, VEGF, survivin and Bcl-2 in MDA-MB-231 cells. Fig 4B demonstrates a substantial decline in the expression of cyclin D1, VEGF, survivin and Bcl-2 proteins in the time-dependent manner.

### CIP suppresses CXCL12-induced MDA-MB-231 Cell invasion

**Fig 5:**
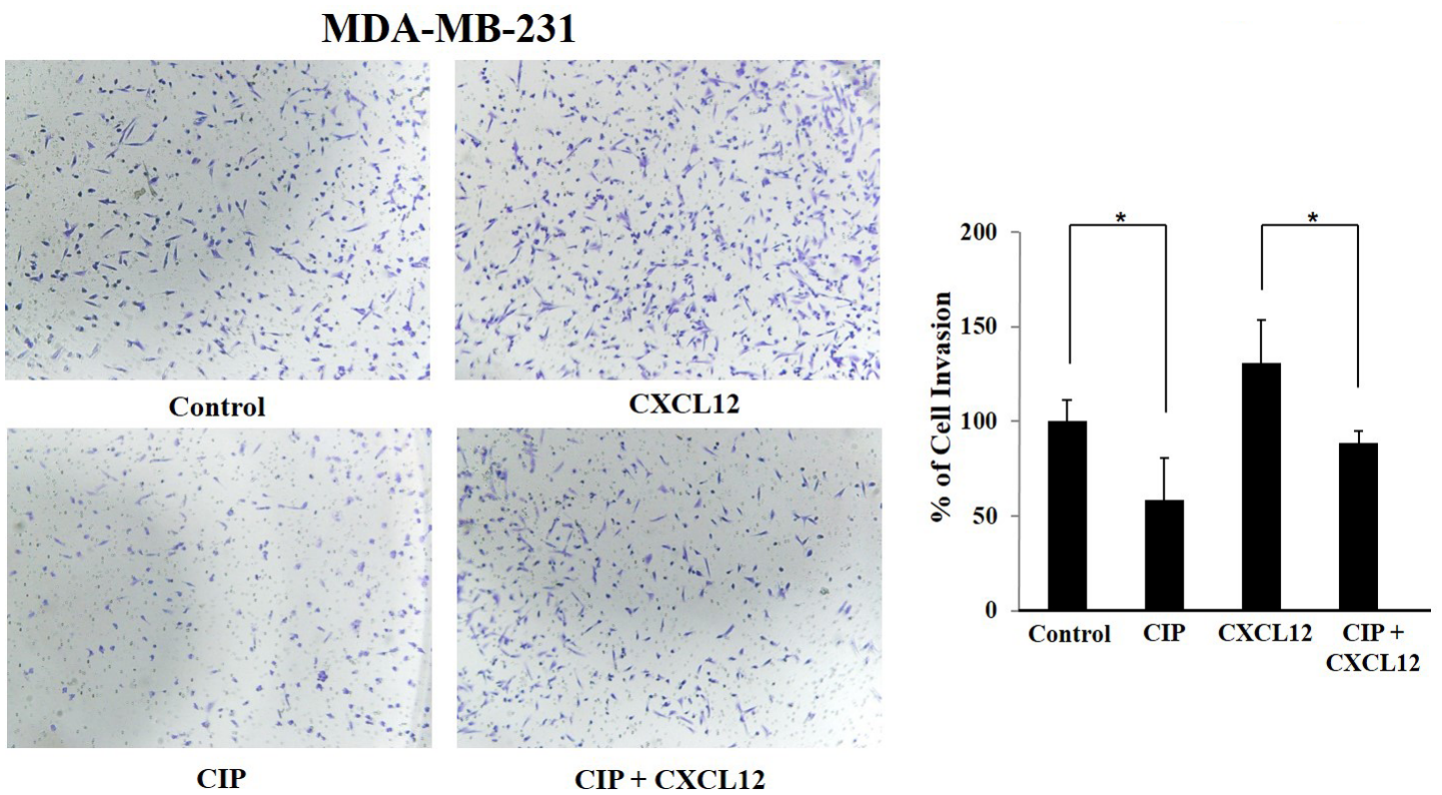
MDA-MB-231 cells were suspended in serum-free DMEM and seeded into the Matrigel transwell chambers and were incubated with CIP (25 μM) for 8 h. The invading cells were fixed and stained with crystal violet solution and invaded cells were counted in five randomly selected areas. CIP significantly suppressed the cell invasion both in the presence and absence of CXCL12 suggesting that CIP interferes with invasive potential of breast cancer cells.

Akt-targeted gene products are known to be involved in motility of cancer cells [5]. Therefore, we evaluated potential of CIP to modulate on cellular invasion according to the method described previously [51]. In addition, several studies have demonstrated the critical role and interaction of CXCL12 with CXCR4 in cancer cells which contributes largely to tumor metastasis [52]. Fig 5 indicates the movement of the cells in the presence and absence of CIP and CXCL12. CIP was found to significantly suppress the invasion of MDA-MB-231 cells by nearly 50%, both in the presence and absence of CXCL12, compared with the respective controls, suggesting that CIP interferes with invasive ability of breast cancer cells.

### Molecular docking analysis

Based on the *in silico* and *in vitro* results, we investigated the binding of the imidazole series compounds to the RAC-Beta Serine/Threonine-Protein Kinase (Akt2 kinase) using MOE platform. Our docking calculations revealed that trisubstituted imidazoles bind with high docking scores:the average of the MOE scores for the compounds is found to be -8.38. To validate our in silico docking results, we have docked previously reported compounds that have low micro molar affinity for Akt2 Kinase (92 molecules having IC_50_ value of 1–50 μM extracted from ChEMBL database) using the same docking protocol. The average of the docking scores for this data is -8.62 which is very close to the average score for the imidazole series.

**Fig 6:**
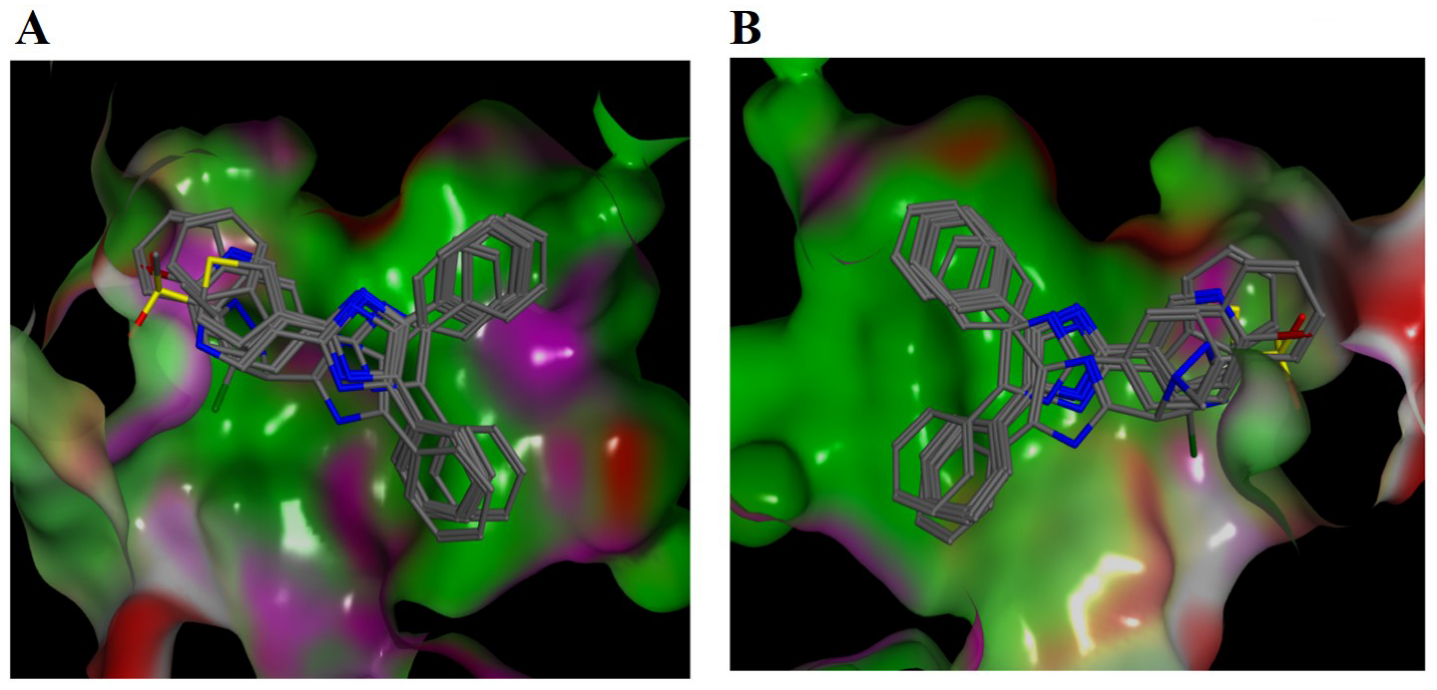
*In silico* molecular docking studies of trisubstituted imidazoles against kinase domain of Akt2: Common binding poses of trisubstituted imidazoles towards the Akt2 kinase domain. The molecular surface of the protein is represented based on the surface polarity; green, pink and red colours show hydrophobic, polar and solvent exposed regions, respectively. For the sake of better visualisation of the binding pocket surface of Akt2, molecular surface was rendered in two panels (A and B) in which ligands were rotated 180 degrees.

Docking of the trisubstituted imidazoles reveal common binding modes as shown in Fig 6. Phenyl moieties at positions 4 and 5 perfectly fit in the hydrophobic profile of the surface of the deep binding pocket (Fig 6A and 6B). Fragments at position 2, which contain electronegative atoms, match to the polar surface of the binding pocket (Fig 6A and 6B).

### Statistical analysis

Student t-test was used to analyze the data. * for p<0.05 was considered statistical significant. * for p<0.05; ** for p<0.005.

## Discussion

The present report demonstrates the anticancer activity of trisubstituted imidazole that targets PI3K/Akt/mTOR pathway. CIP, the lead molecule among the synthesized imidazole derivatives supressed the proliferation of breast cancer (MDA-MB-231, MCF-7) and hepatocellular carcinoma (HepG2) cells and did not induce significant cytotoxicity against normal (MCF-10A, LO2) cells. CIP caused the accumulation of breast cancer cells in Sub-G1 phase, increased the executioner caspase-3/7 activity, downregulated the phosphorylation of PDK, Akt and mTOR in breast cancer cells. Further, it modulated the levels of various proteins involved in cell cycle regulation (Cyclin D1), angiogenesis (VEGF), apoptosis (Caspase-3, PARP), and survival (Survivin, Bcl-2). Further, CIP inhibited the cell invasion indicating its inhibitory role in cell motility.

One of the major events in apoptosis is the activation of caspases thereby cleavage of PARP and activation of caspase-activated deoxyribonuclease (CAD). In the event of cell undergoing apoptosis, caspase-3 activates CAD, and CAD in turn degrades genomic DNA into oligomers [53, 54]. The cells with lesser DNA content are often termed as hypodiploid cells which are detected as Sub-G1 cell population. The lead compound CIP caused the accumulation of cells in Sub-G1 phase demonstrating that CIP induce caspase mediated apoptosis. These results were confirmed and correlated by the cleavage of PARP, a prominent DNA repair enzyme and activation of executioner caspase-3 and caspase-7.

We performed cheminformatics-based target enrichment studies to predict the probable molecular therapeutic target of the lead compound and found a number of targets to be enriched, including Akt protein, and other proteins belonging to the MAPK pathway. Although, the results of the study presented multiple targets, we analysed the effect of CIP on PI3K/Akt/mTOR signalling pathway, because, previous studies have highlighted imidazole derivatives as inhibitors of PI3K/Akt/mTOR proteins [55–57]. In silico predicted target was experimentally validated by in vitro investigation in breast cancer cell lines. Several studies have showed that Akt pathway plays pivotal role in cell survival, growth and proliferation [58]. In order to demonstrate the effect of CIP on Akt signalling pathway, we investigated the effect of CIP on the phosphorylation of Akt pathway proteins including PDK1 (Ser-241), Akt (Ser-473) and mTOR. We observed the downregulation in the phosphorylation of proteins involved in Akt signalling cascade. Therefore, it is evident that, CIP mediates its anticancer activity at least partly *via* blockade of PI3K/Akt/mTOR signalling pathway. This finding opens an avenue for the development of novel trisubstituted imidazole based small molecules as therapeutic agents that target PI3K/Akt/mTOR signalling pathway in human diseases.

## Conclusion

In conclusion, we synthesized trisubstituted imidazoles, identified the bioactive cytotoxic lead structure, predicted the likely target and demonstrated *in vitro* efficacy of lead compound to abrogate the activation of the PI3K/Akt/mTOR pathway in human breast cancer cells. Therefore, designing of CIP-based small molecule inhibitors to abrogate PI3K/Akt/mTOR pathway may serve as an effective therapeutic strategy to fight against various types of cancer.

## Supporting Information

S1 Table Physical parameters and cytotoxicity profile of newly synthesized compounds. (DOC)

S2 Table Top 20 ranked targets for the imidazole series. (DOC)
