# Supplementary material for: Trisubstituted-Imidazoles Induce Apoptosis in Human Breast Cancer Cells by Targeting the Oncogenic PI3K/Akt/mTOR Signaling Pathway
Source: PLoS One. 2016 Apr 20;11(4):e0153155. doi: 10.1371/journal.pone.0153155 (PMC4838272; doi:10.1371/journal.pone.0153155)
Supplement: S1 Table — (DOC) [file pone.0153155.s001.doc]

**S1 Table**

**Supplementary Table 1.** Physical parameters and cytotoxicity profile of newly synthesized compounds

| **Entry** | **2 (a-j)**  **(R)** | **3(a-j)**  **Product** | **Time (h)** | **Yield (%)** | **IC_50_ (µM)** | | | |
| --- | --- | --- | --- | --- | --- | --- | --- | --- |
|  |  |  |  |  | **MDA-MB-231** | **MCF-10A** | **HepG2** | **LO2** |
| **1** | **2a** | **3a** | **7** | **89^a^** | **21.1** | **39.3** | **42.9** | **28.9** |
| **2** | **2b** | **3b** | **7** | **93^b^** | **17.8** | **36.8** | **26.7** | **22** |
| **3** | **2c** | **3c** | **5** | **90^b^** | **33.5** | **33.1** | **28.3** | **> 50** |
| **4** | **2d** | **3d** | **8** | **85^a^** | **> 50** | **> 50** | **48.3** | **> 50** |
| **5** | **2e** | **3e** | **7** | **82^a^** | **31.4** | **31.8** | **29.3** | **34.7** |
| **6** | **2f** | **3f** | **7** | **80^a^** | **26.1** | **39.5** | **39.9** | **35.7** |
| **7** | **2g** | **3g** | **6** | **85^a^** | **43.8** | **> 50** | **> 50** | **27.3** |
| **8** | **2h** | **3h** | **6** | **80^a^** | **26.0** | **> 50** | **48.1** | **27** |
| **9** | **2i** | **3i** | **7** | **90^b^** | **24.1** | **> 50** | **19.7** | **> 50** |
| **10** | **2j** | **3j** | **7** | **95^b^** | **36.8** | **> 50** | **> 50** | **> 50** |
| **11** | **2k** | **3k** | **6** | **85^a^** | **> 50** | **> 50** | **> 50** | **> 50** |

**^a^**Literature reported compounds; **^b^**Novel compounds.
