# Supplementary material for: Trisubstituted-Imidazoles Induce Apoptosis in Human Breast Cancer Cells by Targeting the Oncogenic PI3K/Akt/mTOR Signaling Pathway
Source: PLoS One. 2016 Apr 20;11(4):e0153155. doi: 10.1371/journal.pone.0153155 (PMC4838272; doi:10.1371/journal.pone.0153155)
Supplement: S2 Table — (DOC) [file pone.0153155.s002.doc]

**S2 Table**

**Supplementary table**. Top 20 ranked targets for the Imidazole series.

| Rank | Name | Average Ratio |
| --- | --- | --- |
| 1 | TGF-Beta Receptor Type-1 | 0.0028 |
| **2** | **RAC-Beta Serine/Threonine-Protein Kinase** | **0.0053** |
| 3 | Adenosine Receptor A2b | 0.0060 |
| 4 | Prostaglandin G/H Synthase 1 | 0.0073 |
| 5 | Mitogen-Activated Protein Kinase 12 | 0.0086 |
| 6 | Serine/Threonine-Protein Kinase Chk2 | 0.0093 |
| 7 | Glucagon Receptor | 0.0095 |
| **8** | **RAC-Alpha Serine/Threonine-Protein Kinase** | **0.0108** |
| 9 | Arachidonate 5-Lipoxygenase | 0.0110 |
| 10 | Prostaglandin G/H Synthase 2 | 0.0113 |
| 11 | Casein Kinase I Isoform Delta | 0.0116 |
| 12 | Glycogen Synthase Kinase-3 Beta | 0.0118 |
| 13 | Adenosine Receptor A2a | 0.0120 |
| 14 | Mitogen-Activated Protein Kinase 14 | 0.0127 |
| 15 | Camp-Specific 3',5'-Cyclic Phosphodiesterase 4A | 0.0130 |
| 16 | Serine/Threonine-Protein Kinase B-Raf | 0.0140 |
| 17 | Mitogen-Activated Protein Kinase 11 | 0.0143 |
| 18 | Mitogen-Activated Protein Kinase 9 | 0.0155 |
| 19 | Cell Division Cycle 7-Related Protein Kinase | 0.0155 |
| 20 | Serine/Threonine-Protein Kinase Pim-2 | 0.0170 |
